# Defining critical illness using immunological endotypes in patients with and without sepsis: a cohort study

**DOI:** 10.1186/s13054-023-04571-x

**Published:** 2023-07-20

**Authors:** Jeremy A. Balch, Uan-I Chen, Oliver Liesenfeld, Petr Starostik, Tyler J. Loftus, Philip A. Efron, Scott C. Brakenridge, Timothy E. Sweeney, Lyle L. Moldawer

**Affiliations:** 1 Sepsis and Critical Illness Research Center, Department of Surgery, Shands Hospital, University of Florida College of Medicine, Room 6116, 1600 SW Archer Road, P. O. Box 100019, Gainesville, FL 32610-0019 USA; 2 Inflammatix, Inc., Sunnyvale, CA 94085 USA; 3 UF Health Medical Laboratory at Rocky Point, Department of Pathology, Immunology and Laboratory Medicine, University of Florida College of Medicine, Gainesville, FL 32610 USA; 4 Department of Surgery, Harborview Medical Center, University of Washington School of Medicine, Seattle, WA 63110 USA

**Keywords:** Transcriptomics, Biomarkers, ICU, Outcomes, Sepsis

## Abstract

**Background:**

Sepsis is a heterogenous syndrome with limited therapeutic options. Identifying immunological endotypes through gene expression patterns in septic patients may lead to targeted interventions. We investigated whether patients admitted to a surgical intensive care unit (ICU) with sepsis and with high risk of mortality express similar endotypes to non-septic, but still critically ill patients using two multiplex transcriptomic metrics obtained both on admission to a surgical ICU and at set intervals.

**Methods:**

We analyzed transcriptomic data from 522 patients in two single-site, prospective, observational cohorts admitted to surgical ICUs over a 5-year period ending in July 2020. Using an FDA-cleared analytical platform (nCounter FLEX^®^, NanoString, Inc.), we assessed a previously validated 29-messenger RNA transcriptomic classifier for likelihood of 30-day mortality (IMX-SEV-3) and a 33-messenger RNA transcriptomic endotype classifier. Clinical outcomes included all-cause mortality, development of chronic critical illness, and secondary infections. Univariate and multivariate analyses were performed to assess for true effect and confounding.

**Results:**

Sepsis was associated with a significantly higher predicted and actual hospital mortality. At enrollment, the predominant endotype for both septic and non-septic patients was *adaptive*, though with significantly different distributions. *Inflammopathic* and *coagulopathic* septic patients, as well as *inflammopathic* non-septic patients, showed significantly higher frequencies of secondary infections compared to those with adaptive endotypes (*p *< 0.01). Endotypes changed during ICU hospitalization in 57.5% of patients. Patients who remained *adaptive* had overall better prognosis, while those who remained *inflammopathic* or *coagulopathic* had worse overall outcomes. For severity metrics, patients admitted with sepsis and a high predicted likelihood of mortality showed an *inflammopathic* (49.6%) endotype and had higher rates of cumulative adverse outcomes (67.4%). Patients at low mortality risk, whether septic or non-septic, almost uniformly presented with an adaptive endotype (100% and 93.4%, respectively).

**Conclusion:**

Critically ill surgical patients express different and evolving immunological endotypes depending upon both their sepsis status and severity of their clinical course. Future studies will elucidate whether endotyping critically ill, septic patients can identify individuals for targeted therapeutic interventions to improve patient management and outcomes.

**Supplementary Information:**

The online version contains supplementary material available at 10.1186/s13054-023-04571-x.

## Introduction

Sepsis remains one of the most common causes of mortality and morbidity in critically ill patients, affecting as many as 50 million individuals annually with case mortality rates as high as 40% [1]. Earlier recognition and near-universal implementation of sepsis protocols have improved in-hospital clinical outcomes; however, targeted therapies remain elusive [2–4].

Sepsis is defined as a dysregulated host immune response to infection resulting in life-threatening organ dysfunction [5, 6]. However, inherent to this definition is a wide range of insults and trajectories of physiologic decline. This disease heterogeneity likely explains the lack of efficacy in previous randomized controlled trials employing immune modulating therapeutics [7–11]. To address this heterogeneity, efforts have been made to classify patients based on constellations of observable characteristics and commonly available laboratory values, also called phenotypes [12–15]. However, phenotypes based on these clinical variables may not accurately discriminate differences in the underlying disease mechanisms, also called endotypes. Thus, efforts at phenotyping have not led to substantial changes in patient care or outcomes [16, 17].

Multiplex metabolomics, proteomics, and transcriptomics offer the potential to reveal a spectrum of sepsis endotypes, both illuminating common underlying mechanisms for immunological dyscrasia and providing potential therapeutic targets. Semantically, we choose the term “endotype” to highlight subphenotypes with distinct functional or pathobiological mechanisms amenable to targeted interventions and to contrast against clinically observable phenotypes. While the present classification schema has not been proven to be linked to treatment effect, it does align with previous research by our group and others [7, 18–20]. Previous research has identified 2–5 endotypes in diagnosed sepsis, though they vary with regards to domains, data sources, classification algorithms, statistical methodology, duration of observations, and stated goals [18, 21–26]. Cumulatively, however, these studies have sparked interest in re-defining aspects of critical illness in terms of underlying physiologic perturbations rather than phenotypic syndromes [9, 27].

In this study, we apply 29- and 33-gene transcriptomic signatures to simultaneously classify severity and endotype, respectively, within both septic and non-septic critically ill patients [25, 28–30]. These transcriptomic signatures were originally validated in non-surgical patients with bacterial or viral sepsis, and were classified into *adaptative*, *inflammopathic*, and *coagulopathic* endotypes based on gene ontology analysis [25]. We investigate whether patients admitted to a surgical ICU with sepsis and with high risk of mortality would express similar endotypes to non-septic, but still critically ill patients at-risk of developing sepsis. We hypothesize that patients admitted to a surgical ICU with sepsis and with high risk of mortality would express similar endotypes to non-septic, but still critically ill patients. In addition, we compare differences in endotype on admission between patients with predicted high severity by the transcriptomic metric versus the ground truth of those who clinically developed adverse outcomes. We also examine how these endotypes evolve over time in critically ill patients, tracking those who either rapidly recover versus those who experience adverse outcomes–defined as all-cause (in-hospital, 30-, 90-day) mortality, development or absence of chronic critical illness (CCI), secondary infections, and poor discharge disposition.

## Materials and methods

### Study designs

**Fig. 1:**
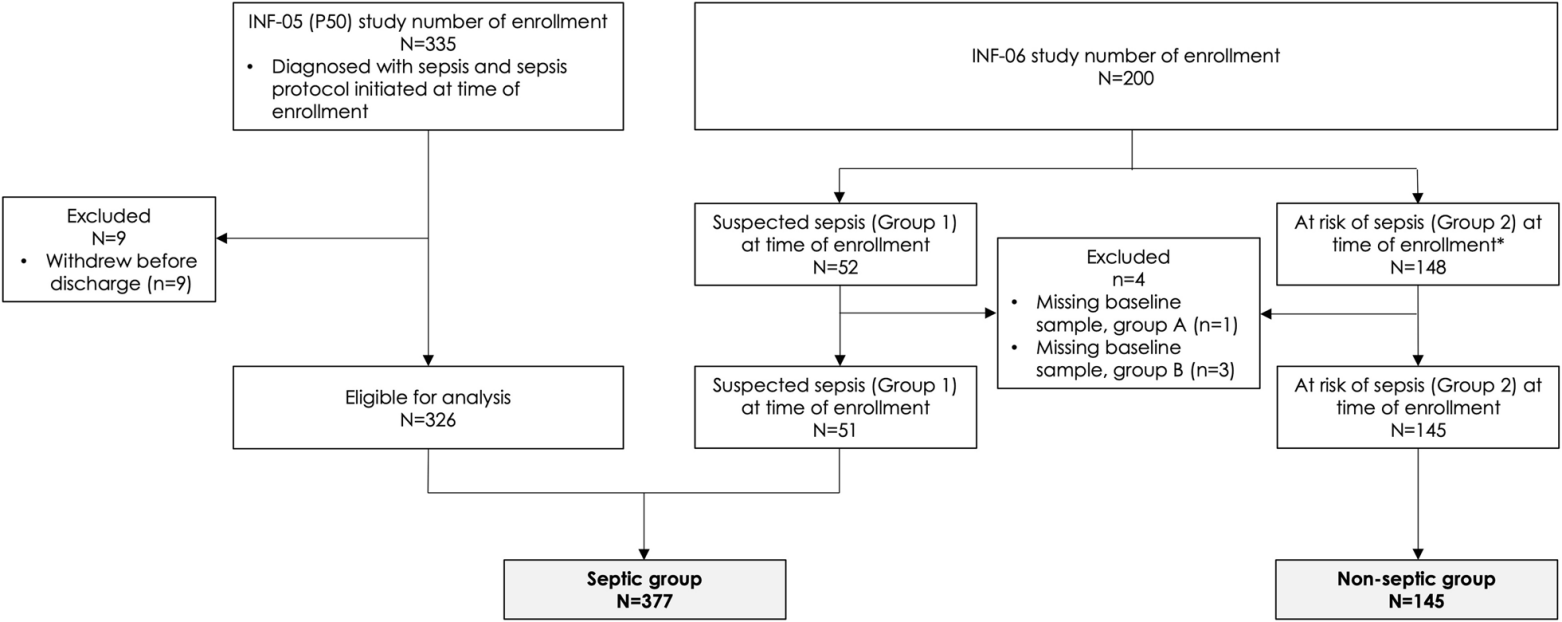
Study inclusion criteria. Study population was derived from two single-site, prospective, observational studies that enrolled a total of 522 patients admitted to a non-cardiac, surgical ICUs. *All data points are at time of enrollment. Therefore, the 11 crossover patients were included in the non-septic group since they were not septic at the time of enrollment

This post hoc study performed transcriptomic analyses on samples from two single-site, prospective, observational cohorts that enrolled a total of 522 patients admitted to non-cardiac, surgical ICUs and were classified as either (1) critically ill patients with a diagnosis of sepsis (septic) or (2) non-septic critically ill patients, at high risk of subsequently developing sepsis (at risk or non-septic; Fig. 1) [28, 29]. As a post hoc analysis, it was not powered for any specific outcome. Data and additional samples were obtained from the University of Florida CTSA Biorepository, a resource available to the scientific community [31]. In the first study (INF-05) [29], the parent cohort included 363 patients admitted to a surgical ICU between January 2015 and January 2020 with a diagnosis of sepsis (NCT02276417). Sepsis cohort inclusion criteria were: (1) age greater than or equal to 18 years, (2) clinical diagnosis of sepsis as defined by 2001 consensus guidelines, and (3) entrance into the electronic health record (EHR)-based sepsis clinical management protocol. Although prospectively enrolled using 2001 sepsis consensus criteria, these patients were retrospectively re-adjudicated and reclassified using Sepsis-3 consensus definitions [5, 32]. Detailed descriptions of the inclusion and exclusion criteria are contained in Additional file 1: Supplemental Materials: Methods.

The second prospective diagnostic and prognostic study (INF-06) was conducted between July 2020 and July 2021 [28] and enrolled two cohorts of critically ill patients at the time of surgical ICU admission (NCT04414189). Comparisons between INF-05 and INF-06 are shown in Additional file 1: Table S1. One cohort included patients with a suspected diagnosis of sepsis admitted to the ICU for protocolized sepsis management, as in the aforementioned study. Sepsis was defined according to Sepsis-3 criteria. The second cohort included critically ill patients without sepsis (e.g., severely injured trauma patients, post-operative patients, patients admitted directly to ICU from emergency department, see Additional file 1: Table S2). Inclusion and exclusion criteria, study design, and cohort flow are contained in Fig. 1 with a more detailed flow diagram shown in Additional file 1: Fig. S1.

In both studies, all enrolled subjects underwent post hoc adjudication by physician-investigators within one week of cohort enrollment to confirm sepsis diagnosis, severity, and source. Hospital-acquired secondary infections were adjudicated by physician-investigators during primary data/chart review utilizing current United States Centers for Disease Control definitions and guidelines [10].

Individual clinical outcome variables included all-cause (in-hospital, 30-, 90-day) mortality, development or absence of chronic critical illness (CCI), secondary infections, and poor discharge disposition. Inpatient clinical trajectory was defined as “early death,” “rapid recovery,” or “CCI.” CCI was defined as an ICU length of stay greater than or equal to 14 days with evidence of persistent organ dysfunction (SOFA score ≥ 2) [33, 34]. Hospitalized patients who died after an ICU length of stay greater than 14 days from the index hospitalization were also classified as CCI. Rapid recovery patients were those discharged from the ICU within 14 days following resolution of organ dysfunction. Poor disposition was defined as discharge to a skilled nursing facility, long-term acute care facility, or hospice. Our study was performed in accordance with the STROBE guidelines**.**

### Sample collections

Blood samples were collected in PAXgene™ blood RNA tubes within 12–24 h of ICU admission and were stored at  − 80 °C for subsequent analysis. In the second study, additional blood samples were also collected on ICU days 4 and 7 and weekly thereafter during ICU stay (Additional file 1: Methods). RNA was extracted with the RNeasy^®^ Plus Micro Kit (QIAGEN, Germantown, MD). The IMX-SEV-3 severity and the 33-mRNA endotyping classifiers were quantitated simultaneously from 200 ng of RNA input using the 510(k)-cleared NanoString nCounter FLEX™ profiler (NanoString, Seattle, WA) according to a validated standard operating protocol in a Clinical Laboratory Improvement Amendments-certified diagnostic laboratory (UF Health Medical Laboratories at Rocky Point, Gainesville, FL) by licensed laboratory technicians.

### Severity and endotype classification

The probability of an adverse clinical outcome (in-hospital, 30-, and 90-day mortality, development of CCI and discharge disposition) was estimated by a 29 host-messenger RNA (mRNA) test (IMX-SEV-3, Inflammatix Inc., Sunnyvale, CA) that uses a machine learning algorithm to report results as both a continuous variable and stratified “risk bands” to meet clinically actionable performance thresholds: “low,” “moderate,” and “high” likelihood of 30-day mortality (see Additional file 1: Fig. S2) [35]. Severity classification was performed using supervised multi-layer perceptron (MLP) models as previously described [30].

Classification into three endotypes was computed from the whole blood expression of 33 host immune mRNAs using a previously published classifier [25, 28, 36]. These endotypes—*adaptive*, *inflammopathic*, and *coagulopathic*—were derived from the difference of geometric means of gene expression for each of three modules. The inflammopathic module comprises the expression of *ARG1*, *LCN2*, *LTF*, *OLFM4*, and *HLA-DMB*; the coagulopathic module comprises *KCNMB4*, *CRISP2*, *HTRA1*, *PPL*, *RHBDF2*, *ZCCHC4*, *YKT6*, *DDX6*, *SENP5*, *RAPGEF1*, *DTX2*, and *RELB*, and the adaptive module comprises *YKT6, PDE4B, TWISTNB, BTN2A2, ZBTB33, PSMB9, CAMK4, TMEM19, SLC12A7, TP53BP1, PLEKHO1, SLC25A22, FRS2, GADD45A, CD24, S100A12,* and *STX1A* expression*.* An overall endotype assignment for each subject was calculated using a 3-class logistic regression model which takes as input the three modules and generates a probability of endotype assignment {for each subject, the total probability [p(Inflammopathic) + p(Adaptive) + p(Coagulopathic)] sums to 1}. Each sample is assigned an endotype according to the highest probability. Numerical values are presented in the Additional file 1: Table S2 [25].

Total leukocyte and absolute lymphocyte counts (ALCs) were determined at the University of Florida Health Clinical and Diagnostic Laboratories. Plasma IL-6 levels were determined using the Luminex MagPix^®^ platform (Austin, TX).

### Statistical analysis

Descriptive data are presented as frequencies and percentages or means and standard deviations (SD). The Fisher exact test or Pearson's Chi-squared test and t-test were used for comparison of categorical and continuous variables, respectively. All significance tests were two sided, with a raw *p *≤ 0.05 considered statistically significant. Significance levels 0.05 > *p *> 0.01 are reported precisely; 0.01 > *p *> 0.001 are reported as ‘*p *< 0.01,’ and lower values are all reported as ‘*p *< 0.001’. Univariable and multivariable logistic regressions were performed, controlling for age, sex, WBC, IL-6, SOFA, endotype, Charlson Comorbidity Index, and septic status. Analyses were performed using the R Project statistical package, version 4.2.0 (R Project for Statistical Computing).

### Study approval

Ethics approvals were obtained from the University of Florida Institutional Review Board (IRB#201400611 and IRB#201702261). Informed consent was obtained from each subject or their surrogate decision-maker. Self-reported or proxy-reported race and ethnicity category data were collected as per National Institutes of Health reporting guidelines and requirements.

## Results

### Septic and non-septic cohorts

The overall analytic cohort consisted of 522 critically ill patients from the two consecutive, prospective observational studies (Fig. 1). Prediction of sepsis severity and endotype analyses were conducted on 377 septic and 145 non-septic patients within 24 h of ICU admission (Table 1). A subset of septic (*N *= 51) and all non-septic (*N *= 145) patients had repeat blood sampling at designated intervals over their ICU stay. Three hundred and twenty-six (86%) septic patients were drawn from the initial cohort (INF-05), and all non-septic patients were drawn from the second cohort (INF-06) [28, 29]. Demographics of included patients are shown in Table 1, while Table 2 shows outcomes, endotypes, and severity predictions of the two critically ill cohorts.

**Table 1:** Patient demographics at enrollment

Variable	At enrollment		*p* value^b^
	Septic (*N *= 377)^a^	Non-septic (*N *= 145)^a^	
Age (yr)	58.9 (15.4)	57.4 (19.4)	0.39
Male	202 (53.6%)	92 (63.5%)	0.04
Race			
African American	38 (10.1%)	10 (7%)	0.02
Asian	2 (0.5%)	0 (0%)	
Other	2 (0.5%)	6 (4.2%)	
White	333 (88.8%)	127 (88.8%)	
Missing	2	2	
WBC (× 1000/mm^3^)	17.9 (8.5)	12.9 (5.5)	< 0.001
Missing	1	6	
Neutrophils (%)	80.7 (12.9)	76.8 (15.3)	0.12
Missing	30	102	
Lymphocytes (%)	5.1 (4)	11.7 (8.1)	< 0.001
Missing	30	102	
Lymphocytes (× 1000/mm^3^)	0.8 (0.5)	1.2 (0.8)	< 0.001
Missing	30	102	
IL-6 (pg/mL)^c^	738.6 (1807)	148.7 (313.7)	< 0.001
Missing	3	2	
SOFA Score	6 (4)	3 (3.2)	< 0.001
Missing	3	0	
Charlson Comorbidity Index	3.2 (2.7)	2.7 (2.5)	0.03
Missing	2	1	

^a^Mean (SD);* n *(%)

^b^Welch two-sample t-test; Pearson's Chi-squared test; Fisher's exact test

^c^values represent samples obtained within 24 h post enrollment

**Table 2:** Clinical outcomes, endotypes, and severity predictions

Variable	Septic (*N *= 377)^a^	Non-septic (*N *= 145)^a^	*p* value^b^
Secondary infection	114 (30.2%)	12 (8.3%)	< 0.001
CCI	122 (32.4%)	10 (6.9%)	< 0.001
Adverse outcome	213 (56.7%)	40 (27.8%)	< 0.001
Missing	1	1	
Poor discharge disposition	152 (40.4%)	23 (16%)	< 0.001
Missing	1	1	
In-hospital mortality	28 (7.4%)	3 (2.1%)	0.02
30-day mortality	38 (10.2%)	6 (4.1%)	0.03
Missing	4	0	
90-day mortality	61 (16.8%)	8 (5.5%)	< 0.001
Missing	13	0	
Endotype			< 0.001
Adaptive	151 (40.1%)	74 (51%)	
Coagulopathic	97 (25.7%)	48 (33.1%)	
Inflammopathic	129 (34.2%)	23 (15.9%)	
IMX-SEV severity risk band			< 0.001
Low	38 (10.1%)	16 (11%)	
Moderate	250 (66.3%)	124 (85.5%)	
High	89 (23.6%)	5 (3.5%)	

*CCI* chronic critical illness, adverse outcome is defined as cumulative incidence of in-hospital, 30-, and 90-day mortality, development of CCI, and poor discharge disposition

^a^n (%)

^b^Pearson's Chi-squared test

As expected, critically ill patients admitted to the ICU with sepsis had significantly higher SOFA and Charlson Comorbidity scores compared to the non-septic cohort, indicating more severe organ dysfunction and greater number of comorbidities. As shown in Table 2, poorer outcomes were observed among the septic cohort, including a higher incidence of secondary infection (30.2 vs. 8.3%, *p *< 0.001), development of CCI (32.4 vs. 6.9%, *p *< 0.001), poor discharge disposition (40.4 vs. 16.0%, *p *< 0.001), in-hospital mortality (7.4 vs. 2.1%, *p *= 0.02), 30-day (10.2 vs. 4.1%, *p *= 0.03), and 90-day (16.8 vs. 5.5%, *p *< 0.01) mortality.

### Endotype distributions and outcomes

Endotype distributions were significantly different between septic and non-septic groups (Table 2). In both septic and non-septic cohorts, the adaptive endotype was most frequent, although it was more common in non-septic patients (40.1% vs. 51%). The inflammopathic endotype was second most common in septic patients and third in non-septic patients (34.2 vs. 15.9%) (Table 2). However, septic patients (*n *= 377) had different clinical outcomes depending upon their endotype at admission (Table 3). Inflammopathic and coagulopathic septic patients had a significantly higher frequency of secondary infections (37% each) compared to septic patients with an adaptive endotype (20%, *p *< 0.01). Similar increases in the frequency of secondary infections were seen in the inflammopathic non-septic patients (26%) versus patients with coagulopathic (4%) or adaptive (5%) endotypes (*p *< 0.01). Thirty-day mortality, CCI, and adverse discharge disposition did not reach statistical significance.

**Table 3:** Endotypes and outcomes on ICU admission

Variable	Septic (*N *= 377)				Non-septic (*N *= 145)			
	Adaptive (*N *= 151)^a^	Coagulopathic (*N *= 97)^a^	Inflammopathic (*N *= 129)^a^	*p* value^b^	Adaptive (*N *= 74)^a^	Coagulopathic (*N *= 48)^a^	Inflammopathic (*N *= 23)^a^	*p* value^b^
Secondary infection	30 (19.9%)	36 (37.1%)	48 (37.2%)	< 0.01	4 (5.4%)	2 (4.2%)	6 (26.1%)	< 0.01
CCI	40 (26.5%)	38 (39.2%)	44 (34.1%)	0.1	2 (2.7%)	2 (4.2%)	6 (26.1%)	< 0.01
Adverse outcome	63 (41.7%)	63 (65.6%)	87 (67.4%)	< 0.001	18 (24.7%)	10 (20.8%)	12 (52.2%)	0.02
Missing	0	1	0		1	0	0	
Poor discharge disposition	49 (32.5%)	42 (43.8%)	61 (47.3%)	0.03	12 (16.4%)	7 (14.6%)	4 (17.4%)	0.95
Missing	0	1	0		1	0	0	
In-hospital mortality	7 (4.6%)	6 (6.2%)	15 (11.6%)	0.07	2 (2.7%)	1 (2.1%)	0 (0%)	> 0.99
30-day mortality	11 (7.4%)	9 (9.5%)	18 (14%)	0.19	4 (5.4%)	1 (2.1%)	1 (4.4%)	0.74
Missing	2	2	0					
90-day mortality	22 (15.2%)	16 (17%)	23 (18.4%)	0.78	5 (6.8%)	2 (4.2%)	1 (4.4%)	0.89
Missing	6	3	4					
IMX-SEV severity risk band				< 0.001				< 0.001
Low	38 (25.2%)	0 (0%)	0 (0.00%)		15 (20.3%)	1 (2.1%)	0 (0%)	
Moderate	113 (74.8%)	72 (74.2%)	65 (50.4%)		59 (79.7%)	47 (97.9%)	18 (78.3%)	
High	0 (0.0%)	25 (25.8%)	64 (49.6%)		0 (0%)	0 (0%)	5 (21.7%)	

*CCI* chronic critical illness, adverse outcome is defined as cumulative incidence of in-hospital, 30-, and 90-day mortality, development of CCI, and poor discharge disposition

^a^Mean (SD); *n* (%)

^b^Kruskal-Wallis rank sum test; Pearson's Chi-squared test; Fisher's exact test

To examine whether endotype at baseline is associated with different patient outcomes, a multivariable logistic regression was conducted by including endotypes and other clinically relevant factors into the model. Of interest, patients with inflammopathic (OR 2.4, 95% CI 1.4–4.1, *p *< 0.001) and coagulopathic endotypes (OR 1.9, 95% CI 1.1–3.1, *p *= 0.014) had higher odds of having an adverse outcome compared to those with the adaptive endotype (Additional file 1: Table S3 and Fig. S3).

### Endotype transitions

**Fig. 2:**
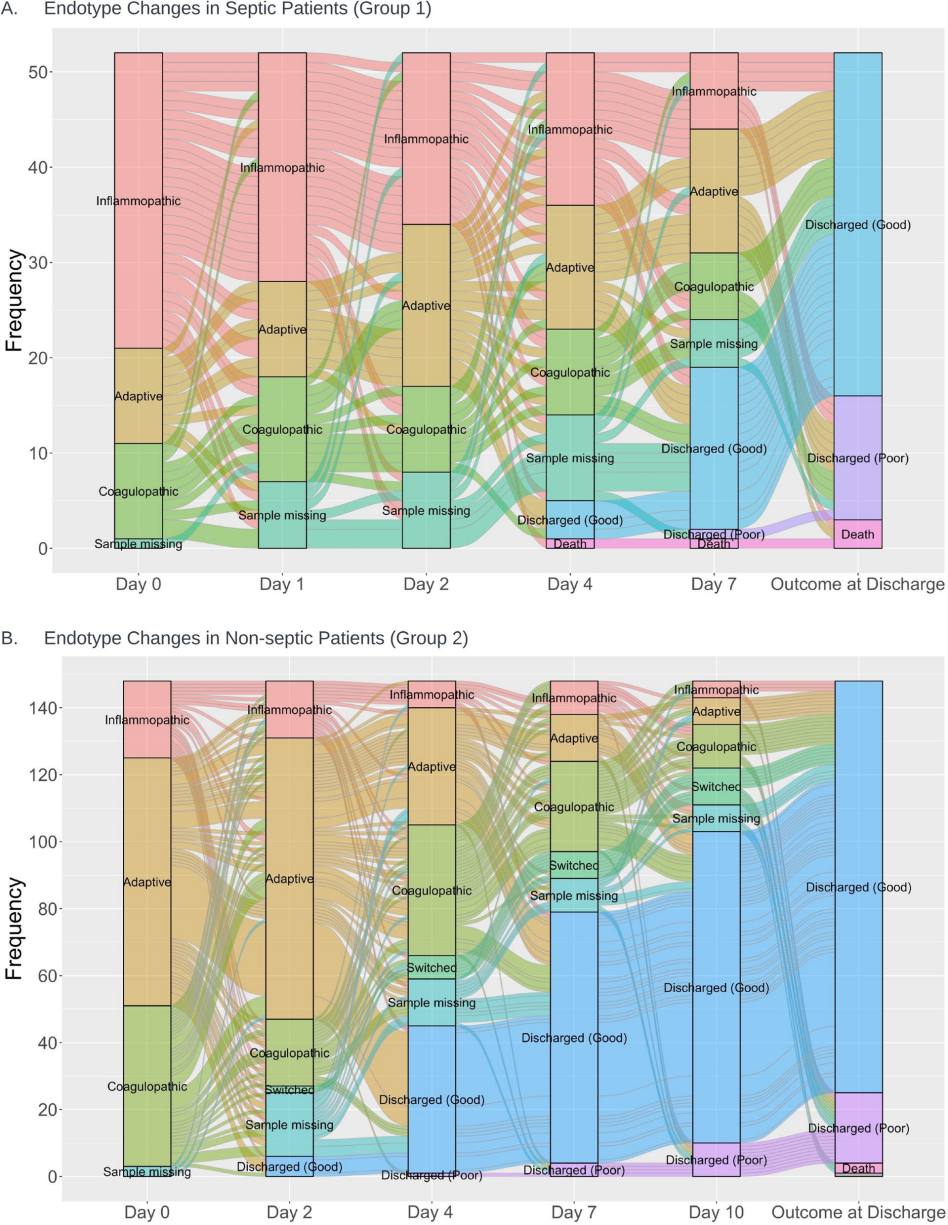
Alluvial Plots of Immunological Endotypes as they Change Over Time in Septic Patients (Group 1) and Non-septic Patients (Group 2). Measurements for both the septic (*n *= 52, Group 1) and non-septic patients (*n *= 145, Group 2) were obtained only from the second cohort (INF-06). 74% of patients changed endotypes during their hospitalization, 19% remained adaptative, 3.5% inflammopathic, and 3.5% coagulopathic. “Switched” is defined as those that transitioned into sepsis

Figure 2 illustrates endotype distributions and transitions over time until death or hospital discharge. Measurements for both the septic (*n *= 52, Group 1) and non-septic patients (*n *= 145, Group 2) were obtained only from the second cohort (INF-06). 61 patients had at least one missing value, with 20% of data missing secondary to declined blood draw and 7% due to inadequate samples, labeling errors, or staff unavailability. Endotypes changed in 57.5% of patients during their hospitalization; of the remaining, 19% remained adaptative, 4% inflammopathic, and 3% coagulopathic.

We assessed pooled outcome data between septic and non-septic patients obtained after the last collected endotype measurement (Table 4). Based on similar clinical behavior and worse overall clinical outcomes, we also pooled inflammopathic and coagulopathic endotypes. In most cases, the final endotype assessment was drawn on day 7 or 10 of ICU admission. There were significant differences in prognosis among the classes depending on endotype trajectory; patients who remained adaptive (*N *= 60) had the best nominal outcomes across all endpoints measured, though these were not individually tested. There were non-significant differences between those who transitioned either to or from adaptive endotype.

**Table 4:** Change in endotypes over time in ICU and subsequent outcomes

Variable	Endotype change				*p* value^e^
	Adaptive to adaptive (*N *= 60)^a^	Adaptive to I/C* (*N *= 24)^b^	I/C to adaptive (*N *= 38)^c^	I/C to I/C (*N *= 74)^d^	
In-hospital mortality	0 (0%)	2 (8.3%)	1 (2.6%)	3 (4.1%)	0.15
30-day mortality	1 (1.7%)	4 (16.7%)	2 (5.3%)	7 (9.5%)	0.06
90-day mortality	2 (3.3%)	5 (20.8%)	3 (7.9%)	9 (12.2%)	0.07
CCI	1 (1.7%)	1 (4.2%)	5 (13.2%)	10 (13.5%)	0.04
Poor discharge disposition	7 (11.7%)	7 (30.4%)	8 (21.1%)	16 (21.9%)	0.2
Missing	0	1	0	1	
Total ICU LOS (days)	2 (1, 4)	6.5 (2, 8)	4 (2, 8)	5 (2, 11)	< 0.001
Missing	0	2	0	0	

^a^First and last endotypes are both adaptive

^b^First endotype was adaptive; last endotype was inflammopathic or coagulopathic

^c^First endotype was inflammopathic or coagulopathic; last endotype was adaptive

^d^First and last endotypes are both inflammopathic and coagulopathic

^e^Fisher’s exact test; Kruskal–Wallis rank sum test

*I/C Inflammopathic/coagulopathic, *ICU* intensive care unit, *LOS* length of stay

### Endotypes and predicted severity

**Fig. 3:**
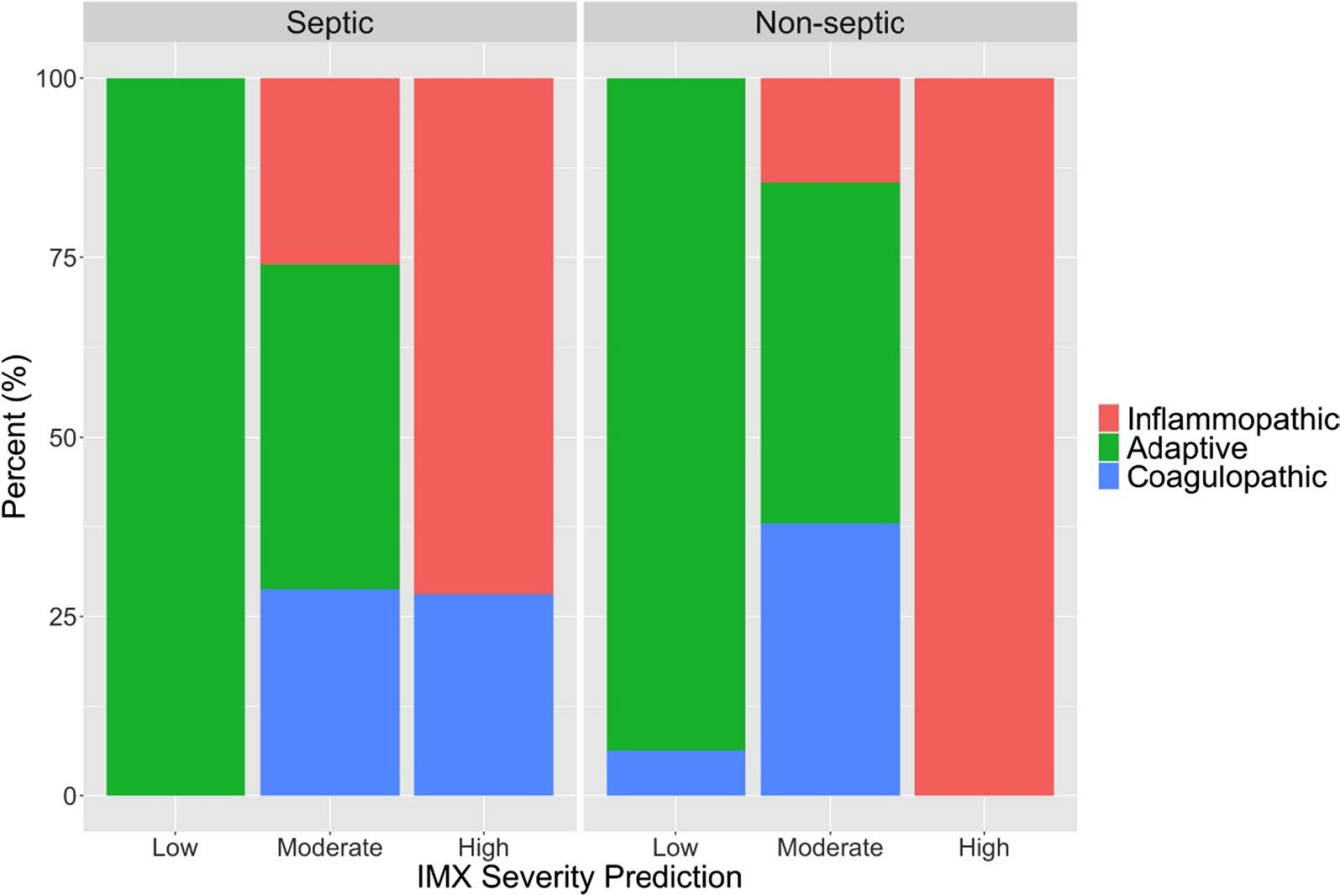
Immunological endotypes of sepsis and at-risk sepsis patients based on their risk of mortality using the IMX-SEV severity prediction model. Sepsis (*N *= 377) and non-septic (*N *= 154) patients were stratified based on their mortality prediction model and assigned to either adaptive, inflammopathic, or coagulopathic. Importantly, patients stratified into the low risk of mortality using the IMX severity index were uniformly adaptive, while patients assigned to high risk of mortality were near uniformly inflammopathic or coagulopathic, regardless of whether they were septic or at risk of sepsis

To better control for disease severity when comparing endotypes, we employed the severity transcriptomic metric (IMX-SEV-3) and found that endotypes were imbalanced across severity metrics (Fig. 3). Patients predicted to be low severity (*n *= 54), independent of their ICU admission cause, were near universally adaptive (98%): only one patient expressed a coagulopathic endotype while the patients with moderate severity prediction by IMX-SEV-3 continued to favor adaptive versus inflammopathic and coagulopathic endotypes (septic cohort: 45 vs. 26 vs. 29%, respectively; non-septic cohort: 48 vs. 15 vs. 38%, respectively). In contrast, those patients with high severity prediction based on IMX-SEV-3 were inflammopathic or coagulopathic in the septic (72% vs. 28%) and inflammopathic in the non-septic (100% vs. 0%) cohorts. We noted that inflammopathic (*n *= 69) and coagulopathic patients (*n *= 25) with a high risk of predicted mortality by IMX-SEV-3 appeared clinically similar, with nonsignificant differences in SOFA score, secondary infection, CCI, adverse outcomes, or mortality. The only noted difference was that inflammopathic patients demonstrated significantly higher plasma IL-6 concentrations than their coagulopathic counterparts (1870 vs. 642 pg/ml, *p *< 0.01; Additional file 1: Table S4).

## Discussion

### Key findings

This post hoc analysis of a septic and non-septic cohorts of critically ill surgical patients showed similar endotype profiles regardless of Sepsis-3 criteria, with an inflammopathic endotype on admission corresponding to high severity and worse outcomes by composite measure. Endotypes transitions occurred frequently during hospital admission. We found no statistically significant differences in mortality.

### Context

Recent advances in sepsis endotyping research benefit from standard comparisons between studies, as advocated by De Merle et al. [7]. By observing endotypes in other septic and non-septic patients, we join efforts to redefine sepsis nosology as a heterogenous condition that shares characteristics across the spectrum of critical illness [9].

Our study shares commonalities and differences with others. The concept of expression-based sepsis subclasses dates to 2009 [37], though the last five years have witnessed an explosion of interest in this field: the MARS consortium investigated and validated patient endotypes in sepsis and identified four groups (MARS1-4) from the expression of 140 genes from 787 septic patients [24]; research by Davenport et al. [38] identified two distinct response signatures reflecting degree of immunosuppression in sepsis patients; and a study by Baghela et al. [18] validated five distinct gene expression profiles across several hospital systems, clustering patients into *neutrophilic-suppressive*, *inflammatory*, *innate host defense*, *interferon*, and *adaptive*. These authors assessed mortality using values drawn at a single time point within 24 h of admission and examined the biological plausibility of the identified genes known to cytokine signaling, cell proliferation, and lymphocyte and metabolic pathways, among others. Selected genes varied between studies, possibly due to differences in cohorts or in the classification techniques used to derive the groups [12, 25]. While these expression profiles carried prognostic significance, these groups did not examine changes in gene expression over the hospital course of illness, nor was there follow-up beyond 14 days.

Several studies, however, have analyzed gene expression profiles at different points during admission and following discharge. In patients expected to require at least 72 h of mechanical ventilation, a follow-up study of the PREVAIL trial assessed samples at days 1, 3, 6, 14, 21, and 28 to differentiate septic and non-septic patients using a novel scoring mechanism [23]. While they demonstrated changes in gene expression profiles through patient admission, they did not analyze outcomes. Similarly, Burnham et al. [39] showed that 46% of patients with community-acquired pneumonia and fecal peritonitis had changes to their gene expression profile on days 1, 3, and 5 of admission. Moreover, while patients who transitioned to the more critically ill group had nominally higher mortality rates, this is was not specifically analyzed. However, Cano-Gamez et al. [40], from the same institution, did demonstrate that patients with the largest decrease in genomic sepsis response had the lowest mortality rate. In a pediatric population, Wong et al. [41] showed that 42% of patients transitioned endotypes, and that those who remained in the more severe class had increased odds of mortality with administration of steroids. Finally, Kwok et al. [19] also examined expression patterns in convalescent samples 6 months after the septic event, finding persistent granulocytic dysfunction.

### Current work

We were able to both identify and track changes in gene expression profile and severity scores over the course of acute critical illness in an adult, critically ill, surgical patient population. While admission endotype appeared to be the strongest predictor of outcomes, the extensive crossover noted between days 2 and 7 suggests that it may be valuable to continue assessing gene expression profiles, rather than focus on a single timepoint. We also noted that very few patients remained inflammatory or coagulopathic throughout their stay, suggesting a transitory maladaptation. This permits monitoring for resolution of immunologic dyscrasia, severity of condition, as well as possible responses to therapy.

Our study recapitulates observations about the 33-mRNA endotypes shown in previous investigations [22, 25, 42]. In contrast with previous investigations, however, we found that inflammopathic and coagulopathic patients had more similarities than differences, perhaps representing a single endotype. When pooled together, we showed that patients who presented with inflammopathic or coagulopathic endotype had increased incidence of adverse outcomes and secondary infections, though differences in in-hospital, 30-day, and 90-day mortality did not reach statistical significance.

In addition to analyzing septic patients, we also included a non-septic, critically ill cohort. While inflammopathic patterns had higher rates of secondary infections regardless of sepsis status, there were no overall changes in mortality or poor discharge disposition. Interestingly, both inflammopathic and coagulopathic subjects in the high-severity risk category had similar outcomes. These results may contribute to the understanding of sepsis as a part of a spectrum of critical illness rather than a separate entity.

Finally, this study applied the endotyping signature in a surgical cohort, while prior evaluations have mostly been in medical, bacterial sepsis, or COVID-19 patients [9, 18, 24, 37, 39, 41]. A recent report suggested the potential for endotypes to underpin different forms of critical illness [9]: a possibility that an *‘inflammopathic’* COVID-19 patient may be similar to an *‘inflammopathic’* surgical sepsis patient in molecular pathophysiology, further contributing to the idea of sepsis as a critical illness subtype.

### Limitations

We note several limitations to our study. First, this study was performed at a single institution with a predominately Caucasian patient population and may lack generalizability. However, both the IMX-SEV-3 severity and the endotyping classifier have been validated multiple times in external hospitals with similar results [25, 36, 42]. Second, our non-septic cohort was broadly defined and with lower overall APACHE II scores. Age, gender, and Charlson comorbidity index were similar between the cohorts. However, when controlling for high-severity risk, we noted similar demographic and patient characteristics between the cohorts. Third, the majority (86%) of septic patients were derived from the initial cohort. These patients generally had higher SOFA scores and rates of CCI, with similar discharge disposition, complications, and mortality to the septic patients recruited in the second cohort. Fourth, as the first cohort was recruited from 2015 until 2020, there is the possibility of data drift, though standard of care for septic patients did not change during that period for our institution. Fifth, the multiple time series population contained only 196 patients, limiting our ability to draw conclusions based on trends and outcomes; and, as common to the literature in transcriptomics, this study is limited as a post-hoc analysis of an existing dataset and may not be powered for a specific outcome, though we have shown significance in several areas. Another important caveat is that with three endotypes, two cohorts, and multiple outcomes measures, we present numerous hypotheses in this manuscript, and we chose not to apply a multiple-hypothesis correction for ease of readership. Larger prospective studies are needed. Sixth, our findings regarding outcomes in final endotype measurements may not be representative of their endotype closer to the outcome measure, as day 10 measurements may have less impact on 30- and 90-day mortality. Finally, this paper did not seek to investigate the biological underpinnings of the mRNAs used in the two classifiers and their relation to pathophysiology; this has been done elsewhere [21, 25].

### Future directions

Results from this study and others could assist in paving the way for personalization of sepsis treatment. By monitoring heterogenous, pathophysiologic responses to therapy, clinicians and researchers may be able to “divide and conquer” the sepsis syndrome and perhaps redefine sepsis along a spectrum of critical illness rather than as a separate entity. Current work into both immunosuppressant and immunostimulant therapies would benefit from targeting specific endotypes. The results of this study may be incorporated into randomized controlled trials or advanced causal analysis techniques employing observational data. From a prognostic standpoint, the conduct of similar endotyping on patients following discharge could also inform our clinical outreach efforts in diverting resources to those with greater follow-up needs.

### Conclusion

Critically ill surgical patients with and without sepsis express different immunological endotypes. These endotypes are dynamic across a patient’s admission and are associated with distinct outcomes, and transitions between them may inform patient prognosis and care. Having identified differences among the patient groups using an endotyping classifier, future prospective studies are needed evaluate differences in therapeutic response between the classes.

## Supplementary Information

**Additional file 1. **Supplemental Materials: Methods.

## Data Availability

The complete raw datasets generated and/or analyzed during the current study are maintained and are available at the UF Clinical and Translational Science Institute Biorepository (https://www.ctsi.ufl.edu/research/laboratory-services/ctsi-biorepository-2/scirc-specimens-archive/). Requests for access to the data are made to the Biorepository directly who will provide a complete deidentified dataset containing both the clinical and transcriptomic data upon request (27).

## Abbreviations

ALC: Absolute lymphocyte count
APACHE II: Acute physiology and chronic health evaluation
CCI: Chronic critical illness
ICU: Intensive care unit
SD: Standard deviation
SOFA: Sequential organ failure assessment
WBC: White blood cell
IL-6: Interleukin-6
