# Supplementary material for: Defining critical illness using immunological endotypes in patients with and without sepsis: a cohort study
Source: Crit Care. 2023 Jul 20;27:292. doi: 10.1186/s13054-023-04571-x (PMC10360294; doi:10.1186/s13054-023-04571-x)
Supplement: Supplementary file 1 — Additional file 1. Supplemental Materials: Methods. [file 13054_2023_4571_MOESM1_ESM.docx]

**Supplemental Materials: Methods**

**INF-05 (NCT02276417)**

This is a secondary analysis in a previously reported cohort from a prospective, observational longitudinal study of hospitalized patients with surgical sepsis. Here, the transcriptomic metric, IMX-SEV-2, was used to predict 30-day mortality when applied to samples obtained from a single-center prospective 1-year longitudinal cohort study of critically ill surgical patients within day 1 following diagnosis of sepsis. The parent cohort included 363 septic patients hospitalized in a 48-bed surgical ICU at a quaternary-care academic hospital between January 2015 and January 2020. Overall cohort inclusion criteria included: 1) age greater than or equal to 18 years, 2) clinical diagnosis of sepsis as defined by 2001 consensus guidelines, and 3) entrance into the electronic medical record (EMR)-based sepsis clinical management protocol. Exclusion criteria included any of the following: 1) refractory shock (death < 24 hr from sepsis protocol initiation) or inability to achieve source control (e.g., total bowel ischemic necrosis), 2) preadmission-expected lifespan less than 3 months, 3) patient/proxy not committed to aggressive management, 4) severe congestive heart failure (New York Heart Association Class IV), 5) Child-Pugh Class C liver disease or pre-liver transplant, 6) known HIV with CD4^+^ count < 200 cells/mm3, 7) patients receiving chronic corticosteroids or immunosuppressive agents, including organ transplant recipients, 8) pregnancy, 9) institutionalized patients, 10) inability to obtain informed consent within 96 hours of enrollment, 11) chemotherapy or radiotherapy within 30 days, 12) severe traumatic brain injury, and 13) spinal cord injury resulting in permanent sensory and/or motor deficits. Informed consent was obtained from each subject or their proxy. Comparisons between this cohort and INF-06 are shown in **Supplementary Table 1.**

| Supplementary Table 1: Comparison of patient cohorts between INF-06 and p50 (INF-05) | | | | |
| --- | --- | --- | --- | --- |
| Variable | N | INF-05  (N = 326)^1^ | INF-06  (N = 200)^1^ | p-value^2^ |
| Age (yr) | 526 | 58.71 (15.44) | 58.24 (18.35) | 0.76 |
| Male | 526 | 173 (53.07%) | 124 (62.00%) | 0.045 |
| Race | 526 |  |  | 0.093 |
| African American |  | 31 (9.51%) | 18 (9.00%) |  |
| Asian |  | 1 (0.31%) | 1 (0.50%) |  |
| Other |  | 2 (0.61%) | 6 (3.00%) |  |
| Unknown |  | 1 (0.31%) | 3 (1.50%) |  |
| White |  | 291 (89.26%) | 172 (86.00%) |  |
| WBC (x1000/mm3) | 515 | 18.07 (8.37) | 13.82 (6.90) | <0.001 |
| Missing |  | 1 | 10 |  |
| Neutrophils (%) | 395 | 79.79 (13.26) | 82.33 (13.26) | 0.1 |
| Missing |  | 26 | 105 |  |
| Lymphocytes (%) | 395 | 4.86 (3.42) | 8.77 (7.10) | <0.001 |
| Missing |  | 26 | 105 |  |
| Lymphocytes (x1000/mm3) | 395 | 0.75 (0.49) | 1.01 (0.67) | <0.001 |
| Missing |  | 26 | 105 |  |
| CRP (mg/L) | 196 | 193.46 (96.81) | 190.49 (110.27) | 0.86 |
| Missing |  | 186 | 144 |  |
| IL-6 (pg/mL) | 413 | 1,102.99 (2,266.88) | 195.98 (385.04) | <0.001 |
| Missing |  | 107 | 6 |  |
| SOFA Score | 522 | 6.27 (3.96) | 3.41 (3.45) | <0.001 |
| Missing |  | 3 | 1 |  |
| Diabetes | 525 | 114 (34.97%) | 53 (26.63%) | 0.047 |
| Missing |  | 0 | 1 |  |
| Secondary Infection | 526 | 105 (32.21%) | 22 (11.00%) | <0.001 |
| CCI | 526 |  |  | <0.001 |
| No |  | 211 (64.72%) | 183 (91.50%) |  |
| Yes |  | 115 (35.28%) | 17 (8.50%) |  |
| Adverse Outcome | 524 | 187 (57.36%) | 68 (34.34%) | <0.001 |
| Missing |  | 0 | 2 |  |
| Discharge Disposition | 524 |  |  | <0.001 |
| GOOD |  | 189 (57.98%) | 159 (80.30%) |  |
| POOR |  | 137 (42.02%) | 39 (19.70%) |  |
| Missing |  | 0 | 2 |  |
| In-Hospital Mortality | 526 | 25 (7.67%) | 6 (3.00%) | 0.027 |
| 30-Day Mortality | 522 | 30 (9.32%) | 15 (7.50%) | 0.47 |
| Missing |  | 4 | 0 |  |
| 3-Month Mortality | 513 | 50 (15.97%) | 20 (10.00%) | 0.055 |
| Missing |  | 13 | 0 |  |
| Death | 501 | 72 (23.92%) | 20 (10.00%) | <0.001 |
| Missing |  | 25 | 0 |  |
| Endotype | 523 |  |  | 0.72 |
| Inflammopathic |  | 98 (30.06%) | 56 (28.43%) |  |
| Adaptive |  | 141 (43.25%) | 82 (41.62%) |  |
| Coagulopathic |  | 87 (26.69%) | 59 (29.95%) |  |
| Missing |  | 0 | 3 |  |
| Charlson Comorbidity Index | 523 | 3.16 (2.65) | 2.91 (2.73) | 0.3 |
| Missing |  | 2 | 1 |  |
| ^1^Mean (SD); n(%) | | | | |
| 2Welch Two Sample t-test; Pearson's Chi-squared test; Fisher's exact test | | | | |

As the parent cohort was designed prior to the publication of Sepsis-3 consensus guidelines, patients were enrolled into the sampling cohort using 2001 sepsis consensus criteria definitions. Subsequently, patients were retrospectively readjudicated for sepsis and septic shock using the Sepsis-3 guidelines.

***Primary Outcomes and Clinical Adjudication***

Discharge disposition was prospectively classified based on known associations with long-term outcomes as either “good” (home with or without healthcare services or rehabilitation facility) or “poor” (long-term acute-care facility), skilled nursing facility, another acute care hospital, hospice, or inpatient death). Individual clinical outcome variables included: 1) 30-day (all-cause) mortality, 2) development or absence of chronic critical illness (CCI), 3) discharge disposition, and 4) secondary infections. Inpatient clinical trajectory was defined as “early death,” “rapid recovery” (RAP), or “CCI.” Early death was defined as death within 14 days of sepsis onset. CCI was defined as an ICU length of stay (LOS) greater than or equal to 14 days with evidence of persistent organ dysfunction based on components of the SOFA score. Hospitalized patients who died after an ICU LOS greater than 14 days from the index hospitalization were classified as CCI. RAP patients were those discharged from the ICU within 14 days with resolution of organ dysfunction. Patients were defined as having an “adverse clinical outcome” if they experienced a secondary infection, CCI, poor discharge disposition, and/or mortality within the first 30 days.

***Sample Collection and Analyses***

Blood samples were collected in PAXgene tubes within 24 hours following initiation of EMR-based sepsis management protocols and were stored at ^–^80°C for subsequent bulk analysis. Total leukocyte counts, absolute lymphocyte counts (ALCs), and C-reactive protein concentrations were determined at the University of Florida Health Clinical and Diagnostic Laboratories.

***INF-06 (***NCT04414189)

This prospective prognostic study enrolled two cohorts of critically ill patients at the time of surgical ICU admission between July 1, 2020, and July 30, 2021. Cohort A included patients with a suspected diagnosis of sepsis admitted to the ICU for protocolized sepsis management. Cohort B included critically ill patients admitted to the ICU without currently suspected sepsis but considered at high risk for subsequent infection (eg, postoperative, severe trauma). Patients in cohort B who subsequently developed sepsis during their ICU course were considered independently as an additional crossover cohort. Inclusion and exclusion criteria, study design, and cohort flow are shown in (**Supplementary Figure 1)**, consistent with Enhancing the Quality and Transparency of Health Research Standards for Reporting of Diagnostic Accuracy (STARD) reporting guidelines. All patients were managed under standardized clinical management protocols.

**Supplementary Figure 1.** Flow Diagram for Study Design.


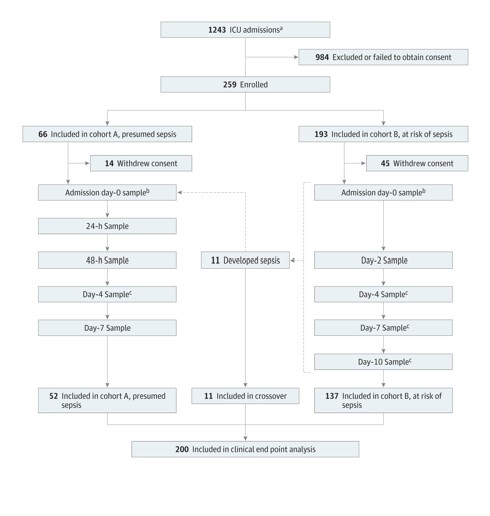


An RNA blood collection tube (PAXgene; Becton Dickinson) and plasma samples for measuring procalcitonin and interleukin 6 (IL-6) levels were collected at the time of enrollment (within 12 hours of ICU admission, labeled as day 0), and on subsequent days depending on the cohort (**Supplementary** **Figure 1**). Self-reported or proxy-reported race and ethnicity category data were collected as per National Institutes of Health reporting guidelines and requirements**.**

Inclusion criteria for Cohort B (“At-Risk”) consisted of intensive care unit (ICU) admission from the emergency department non-trauma, postoperative ICU admission, ICU transfer from the emergency department for severe trauma (patients with Injury Severity Scores >15, hemorrhagic shock, and/or severe chest trauma), and inpatient transfer from ward to ICU (**Supplementary Table 2**). Samples for analysis were obtained within 6 hours.

***Primary Outcomes and Clinical Adjudication***

The primary clinical outcome for IMX-SEV-3 prediction was 30-day mortality, determined via clinical records and telephone follow-up with the patient, their proxy, or their designated contact and cross-checked through the US Social Security Death Index. We compared estimated performance and analyzed temporal trends of IMX classifier scores across the 3 cohorts of critically ill patients: cohort A (suspected sepsis), cohort B (at high risk), and crossover cohort of patients who crossed over from cohort B to cohort A with hospital-acquired sepsis.

**Supplementary Table 2:** Reasons for ICU Admission in Subjects Considered to be At Risk for Developing Sepsis (Cohort B, INF06).

| Diagnosis | Sample Size (n=148) | Percentage |
| --- | --- | --- |
| Trauma | 68 | 45.9% |
| Emergency surgery | 24 | 16.2% |
| Complications associated with cancer | 21 | 14.2% |
| *Clostridium difficile* infection | 19 | 12.8% |
| Vascular disease | 17 | 11.5% |
| Gastrointestinal disease including pancreatitis | 12 | 8.1% |
| Other | 6 | 4.1% |

To aid clinical actionability, Severity scores are stratified into high, moderate, and low-severity interpretation bands (**Supplementary Figure 2**).

**Supplementary Figure 2**: Diagnostic thresholds for likelihood of 28 day mortality. Diagnostic thresholds were set based on prespecified criteria so that the severity scores would be separated into discrete interpretation bands each. The thresholds were originally set in training data targeting likelihood ratios (LRs) of 0.02 in the low bands, and 10 in the upper bands. The selected cutoffs were <0.042, 0.043-0.157, and >0.158.


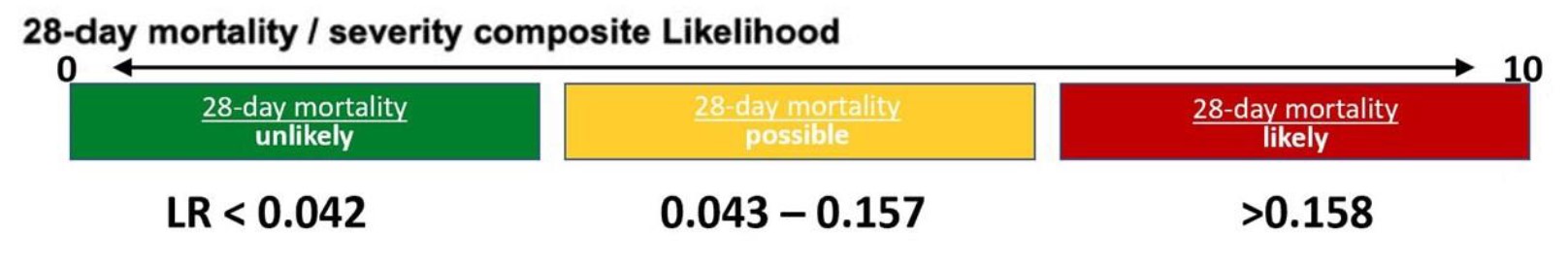


**Supplemental Materials: Additional Results**

**Supplementary Table 3:** Endotype assignments. Overall endotype assignment for each subject was calculated using a 3-class logistic regression model which takes as input the three ‘modules’ and generates a probability of endotype assignment (for each subject, the total probability [p(Inflammopathic) + p(Adaptive) + p(Coagulopathic)] sums to 1). Each sample is assigned an endotype according to the highest probability.

| Variable | Septic (N = 377)^1^ | Non-septic (N = 145)^1^ |
| --- | --- | --- |
| **P(Inflammopathic)** | 0.33 (0.33) | 0.20 (0.25) |
| **P(Adaptive)** | 0.39 (0.44) | 0.49 (0.42) |
| **P(Coagulopathic)** | 0.28 (0.28) | 0.31 (0.30) |
| ^1^Mean (SD) | | |

**Supplementary Figure 3**: Scatterplots demonstrating correlation between endotypes showing distinction between adaptative and non-adaptive groups with less distinction between inflammopathic and coagulopathic groups. However, in previous publications [1], they have repeatedly shown different clinical characteristics, so they were maintained in our present model.


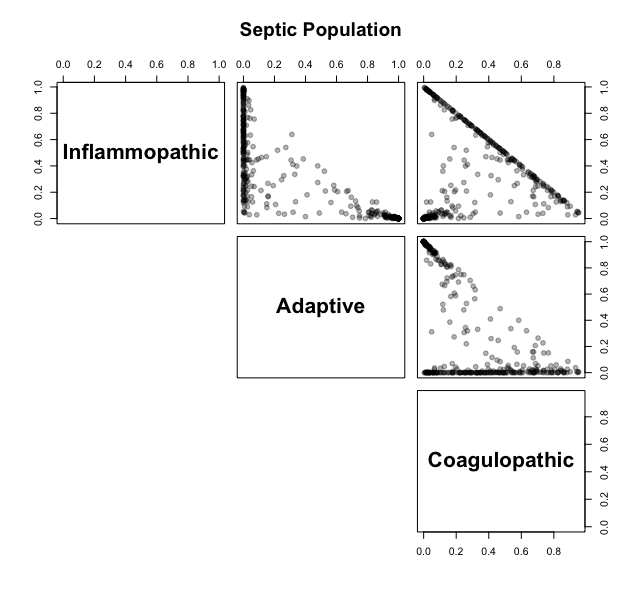

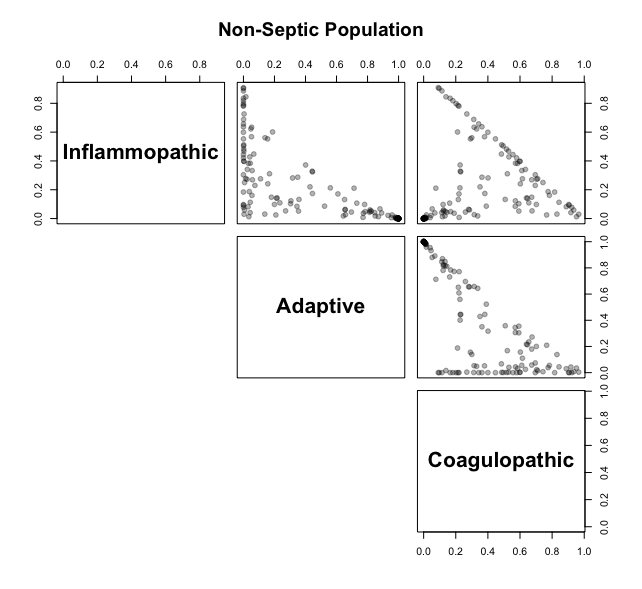


**Supplementary Table 4:** Baseline characteristics and outcomes by endotype among those with high IMX-SEV-3 scores

| Variable | Inflammopathic (N = 69)^1^ | Adaptive (N = 0)^1^ | Coagulopathic (N = 25)^1^ | p-value^2^ |
| --- | --- | --- | --- | --- |
| **Age (yr)** | 62.14 (13.30) | NA (NA) | 65.00 (15.27) | 0.29 |
| **Male** | 37 (53.62%) | 0 (NA%) | 15 (60.00%) | 0.64 |
| **Race** |  |  |  | 0.50 |
| African American | 5 (7.46%) | 0 (NA%) | 1 (4.00%) |  |
| Asian | 1 (1.49%) | 0 (NA%) | 0 (0.00%) |  |
| Other | 0 (0.00%) | 0 (NA%) | 1 (4.00%) |  |
| White | 61 (91.04%) | 0 (NA%) | 23 (92.00%) |  |
| Missing | 2 | 0 | 0 |  |
| **WBC (x1000/mm3)** | 16.51 (8.89) | NA (NA) | 16.92 (9.47) | 0.94 |
| **Neutrophils (%)** | 76.87 (17.31) | NA (NA) | 76.65 (17.01) | 0.81 |
| Missing | 10 | 0 | 4 |  |
| **Lymphocytes (%)** | 4.70 (5.28) | NA (NA) | 4.85 (3.82) | 0.54 |
| Missing | 10 | 0 | 4 |  |
| **Lymphocytes (x1000/mm3)** | 0.55 (0.36) | NA (NA) | 0.79 (0.63) | 0.28 |
| Missing | 10 | 0 | 4 |  |
| **Platelets (x1000/mm3)** |  |  |  | 0.95 |
| >= 150k | 44 (63.77%) | 0 (NA%) | 17 (68.00%) |  |
| < 150k | 12 (17.39%) | 0 (NA%) | 4 (16.00%) |  |
| < 100k | 10 (14.49%) | 0 (NA%) | 4 (16.00%) |  |
| < 50k | 3 (4.35%) | 0 (NA%) | 0 (0.00%) |  |
| < 20k | 0 (0.00%) | 0 (NA%) | 0 (0.00%) |  |
| **CRP (mg/L)** | 175.76 (100.98) | NA (NA) | 234.32 (134.69) | 0.27 |
| Missing | 28 | 0 | 14 |  |
| **IL-6 (pg/mL, within 12hr of enrollment, or 24hr if 12hr samples are not available)** | 1,870.37 (2,815.47) | NA (NA) | 641.64 (1,449.8) | 0.003 |
| Missing | 0 | 0 | 1 |  |
| **SOFA Score** | 8.84 (5.11) | NA (NA) | 7.60 (3.55) | 0.46 |
| **Secondary Infection** | 34 (49.28%) | 0 (NA%) | 11 (44.00%) | 0.82 |
| **CCI** | 33 (47.83%) | 0 (NA%) | 14 (56.00%) | 0.64 |
| **Adverse Outcome** | 60 (86.96%) | 0 (NA%) | 19 (79.17%) | 0.34 |
| Missing | 0 | 0 | 1 |  |
| **Poor Discharge Disposition** | 42 (60.87%) | 0 (NA%) | 15 (62.50%) | >0.99 |
| Missing | 0 | 0 | 1 |  |
| **In-Hospital Mortality** | 12 (17.39%) | 0 (NA%) | 5 (20.00%) | 0.77 |
| **30-Day Mortality** | 16 (23.19%) | 0 (NA%) | 8 (32.00%) | 0.43 |
| **90-Day Mortality** | 20 (29.41%) | 0 (NA%) | 10 (40.00%) | 0.45 |
| Missing | 1 | 0 | 0 |  |
| **Charlson Comorbidity Index** | 3.29 (2.33) | NA (NA) | 4.17 (3.25) | 0.21 |
| Missing | 0 | 0 | 1 |  |
| ^1^Mean (SD); n (%) | | | | |
| ^2^Wilcoxon rank sum test; Fisher's exact test | | | | |

References

1. Sweeney TE, Perumal TM, Henao R, Nichols M, Howrylak JA, Choi AM, Bermejo-Martin JF, Almansa R, Tamayo E, Davenport EE *et al*: **A community approach to mortality prediction in sepsis via gene expression analysis**. *Nat Commun* 2018, **9**(1):694.
